# An internet-based cross-sectional study on infection control practices and drug use for COVID-19 prevention in Nigerian adults

**DOI:** 10.11604/pamj.2022.42.85.31596

**Published:** 2022-06-01

**Authors:** Adedoyin Oyeyimika Ogunyemi, Adedunni Wumi Olusanya, Adesina Paul Arikawe, Oluwarotimi Bolaji Olopade, Uyiekpen Ima-Edomwonyi, Roland Oluwapelumi Ojo

**Affiliations:** 1 Department of Community Medicine, Faculty of Clinical Sciences, College of Medicine, University of Lagos, Idi-Araba, Lagos State, Nigeria,; 2 Department of Pharmacology, Therapeutics & Toxicology, Faculty of Basic Medical Sciences, College of Medicine, University of Lagos, Idi-Araba, Lagos State, Nigeria,; 3 Department of Physiology, Faculty of Basic Medical Sciences, College of Medicine, University of Lagos, Idi-Araba, Lagos State, Nigeria,; 4 Department of Medicine, Lagos University Teaching Hospital, Idi-Araba, Lagos State, Nigeria,; 5 Department of Community Medicine, Lagos University Teaching Hospital, Idi- Araba, Lagos State, Nigeria

**Keywords:** COVID-19, infection control, anti-bacterial agents, masks, pharmaceutical preparations, Ivermectin, antimalarials, plant preparations, Nigeria

## Abstract

**Introduction:**

COVID-19 has affected several millions of people globally and various means have been employed to curb the spread. This nationwide survey investigated adherence to infection control protocols and drug uptake among Nigerian adults.

**Methods:**

this was a descriptive cross-sectional survey using an internet-based questionnaire to investigate adherence to infection control practices and drug use among adults, who have been resident in Nigeria for at least 6 months. The data was analyzed using Stata software version 16 with levels of significance at p<0.05.

**Results:**

a total of 1235 adults participated in the study. The respondents were aged 18-78 years with a mean age of 36.3 ±11.1 years. Over half (53.0%) of the participants were between 31-50 years. The male to female ratio was 1: 1.45. Majority (92.6%) had a minimum of tertiary educational qualification. One hundred (27.1%) reported a positive test result for COVID-19. A total of 1,204 (97.5%) admitted to wearing of face masks, 1,125 (91.1%) washed hands regularly, 1,142 (92.5%) used hand sanitizers while physical distancing was maintained in 985 (79.8%). A total of 854 (69.2%) were on at least a drug or herbal based therapy. Herbal based remedies were used in 112 (9.1%), supplements in 763 (61.8%), antibiotics in 210 (17%), ivermectin in 205 (16.6%), and antimalarials in 128 (10.4%) participants.

**Conclusion:**

adherence to behavioural measures was high among the population, with widespread uptake of supplements, antibiotics, and antimalarial drugs. The high uptake of antibiotics emphasizes the need to step up regulatory policies for antibiotic use.

## Introduction

Coronavirus disease (COVID-19) was first reported in December 2019 following an outbreak in the Wuhan region of China and since then has affected over 200 million people with over 4 million deaths globally as of August 2021 [1].

In Africa, over forty million people have been tested for COVID-19, with about four million positive cases and over 90,000 deaths reported as of August 2021. Nigeria is one of the countries most affected in sub-Saharan Africa with over 167 000 cases since it was first reported in March 2020 [1]. The disease is caused by SARS-CoV-2, an RNA virus which belongs to the genus betacoronavirus. The disease is highly contagious and finding a definitive cure has been elusive, as such measures aimed at reducing the rate of transmission has been employed globally to curb the rate of spread of the disease [2,3]. Different preventive strategies have been employed to prevent COVID-19 infection.

Protocols and policies were developed globally for the control of COVID-19 among which were lockdowns that involved several countries including Nigeria [4]. Other recommendations included the avoidance of social gathering, use of face masks, regular handwashing, self-isolation post-contact etc. and these measures have been shown to mitigate the rates of spread of COVID-19. A study reported that the use of facemasks has an effectiveness of at least 40% if about 50% of the population adopt its use [2]. A mathematical model that used data from COVID-19 transmission dynamics from New York City and the entire US showed that the use of high efficacy masks will lead to a dramatic reduction of COVID-19 burden if at least 70% of the population used facemasks. The study also showed strict social-distancing measures in combination with use of a moderately effective facemasks can help to eliminate the disease if there is at least 10% adherence from the population [5]. For effective control of COVID-19, it is important for the population to adhere strictly to laid-down protocols. Studies have been carried out to assess the levels of adherence and the determinants in different parts of the world with variations in levels across different regions [6-8]. There is paucity of data on adoption of behavioural changes in our environment.

Pharmacological prevention strategies employed for the prevention of COVID-19 include the use of vaccines and post exposure prophylaxis. Currently vaccines are being rolled out on a global scale for emergency use for the protection of infection mainly in the western world. On the other hand, drugs such as chloroquine, hydroxychloroquine, ivermectin are being used for prophylactic purposes against COVID -19 globally despite conflicting evidence of their efficacy. Other drugs used for preventive control include doxycycline, remdesivir, lopinavir/ritonavir, ribavirin and fluoroquinolones [9-11]. In Africa where healthcare facilities are stretched and overwhelmed, people have resorted to several means to prevent COVID-19 infection and these include the use of drugs and herbal based home remedies containing natural spices (turmeric, ginger, garlic etc.) and leaves (neem, paw, guava, etc.) [12,13]. Although some of these herbal based therapies are consumed as food components, there are concerns on the possibility of adverse reactions resulting from drug-drug, drug-food and drug-disease interaction. Therefore, this study aimed to investigate the adherence to infection control practices/behavioural recommendations and use of drugs and other remedies for prevention of COVID-19 among adult Nigerian population.

## Methods

**Data collection tool:** self-administered, standard and valid questionnaire was adapted from similar studies and filled electronically using google survey forms by the respondents.

**Data collection:** section A of the questionnaire contained questions on socio-demographic characteristics; section B asked questions on COVID-19 infections while section C was on drug use for suspected or confirmed infections. Some questions were made compulsory to fill in to ensure completeness. Five researchers were responsible for sending out links to the questionnaires using social media platforms such as WhatsApp and Facebook platforms. To address the potential source of bias, the initial 1321 entries were reduced to 1235 after cleaning and removing multiple entries.

**Sample size:** the sample size was calculated using the formula for descriptive studies


$$n=\frac{z^{2}}{pqd^{2}}$$

Where n was the minimum sample size for a population =10,000; Z was the standard normal deviate corresponding to 95% confidence level (standard value of 1.96). The p, prevalence was from a similar study on prophylactic drug use for the Ebola virus [14] which was 0.71 while d, the margin of error was set at 0.05. Assuming a non-response rate of 10%, a minimum sample size of 348 was calculated to be collected over a duration of 4 weeks but this was exceeded over the study duration.

**Data analysis:** the data was analyzed using Stata software version 16. Frequencies and percentages were generated and represented in tables for sociodemographic variables and prevalence data. After checking for normality, the mean and standard deviation was calculated for the respondents´ ages. Chi-square test was used to test for association for categorical variables to assess socio-demographic characteristics and behavioural patterns. The level of significance was set at p<0.05.

**Ethical approval:** this study was granted ethical approval with number ADM/DCST/HREC/APP/4108 from the Health Research and Ethics Committee of the Lagos University Teaching Hospital. The respondents were asked to signify consent by typing in a box after reading the instructions and eligibility criteria in the first part of the questionnaire. Those who did not consent were asked not to proceed further. The respondents were informed that there was no consequence if they opted out of the study at any time.

## Results

**Sociodemographic analysis**: the study population consisted of 1235 adults with an age range of 18-78 years and a mean age of 36.3 ±11.1 years. Majority (53.0%) of the participants were between 31-50 years. Male to female ratio was 1: 1.45. Other characteristics are shown in Table 1 below. The respondents were distributed across 30 states and the majority were from the epicentres which are Lagos and the FCT, Abuja. From the population studied (Table 2), 35.5% of patients had been exposed to an active case of COVID-19 and 30.1% had a COVID test in the past. Of those who had a test, 27.1% had a positive test.

**Table 1:** demographic characteristics of participants

Variable	Frequency	Percentage
**Age group**		
18-30	455	36.9
31-50	655	53.0
51-70	118	9.6
71-90	7	0.6
**Gender**		
Female	732	59.3
Male	503	40.7
**Educational status**		
Primary	1	0.08
Secondary	91	7.4
Tertiary	597	48.3
Postgraduate	546	44.2
**Employment status**		
Self-employed	761	61.6
Employed	321	26.0
Non-employed	153	12.4

**Table 2:** history of exposure to COVID-19

Variable	Frequency	Percentage
**Contact with COVID-19 patient (active)**		
Yes	438	35.5
No	645	52.2
Maybe	152	12.3
**Symptoms suspicious**		
Maybe	73	5.9
No	664	53.8
Yes	498	40.3
**COVID-19 test**		
No	858	69.9
Yes	370	30.1
**COVID-19 positive tests**		
No	269	72.9
Yes	100	27.1

**Descriptive analysis**: as shown in Table 3, a total of 1,204 (97.5%) admitted to wearing of face masks, 1,125 (91.1%) washed hands regularly, 1,142 (92.5%) used hand sanitizers while physical distancing was maintained in 985 (79.8%). A total of 854 (69.2%) took a drug or herbal based therapy to prevent infection. Herbal based remedies were used in 112 (9.1%), supplements in 763 (61.8%), antibiotics in 210 (17%) patients either in monotherapy or polytherapy, ivermectin in 205 (16.6%), and antimalarial in 128 (10.4%). About two out of three (65.99%) participants in this study took drugs for the prevention of COVID-19 (Table 4) with 24.5% taking a drug, 12.1% taking 2 drugs and 12.96% taking 3 drugs. A total of 7.7% of the population took 5 or more drugs. Modal number of drugs consumed was 9 which was consumed by one participant.

**Table 3:** reported preventive measures among participants

Variable	Frequency	Percentage
Wearing of face masks	1,204	97.5
Regular washing of hands	1,125	91.1
Use of hand sanitizers	1,142	92.5
Physical distancing	985	79.8
Avoidance of social gathering	916	74.2
Fruits	525	42.5
Steam inhalation	398	32.2
Drinking of hot water	390	31.6
Drugs and herbal based remedies	854	69.2
Herbal based remedies	112	9.1
Drugs	833	67.4

**Table 4:** drug use pattern among respondents

Drugs	Frequency	Percentage
Ivermectin	205	16.6
Colchicine	4	0.3
Remdesivir	4	0.3
**Antimalarial**	128	10.4
Chloroquine	85	6.9
Hydroxychloroquine	49	4.0
Other antimalarials	8	0.6
**Antibiotics**	232	18.8
Azithromycin	178	14.4
Ciprofloxacin	30	2.4
Levofloxacin	8	0.6
Other antibiotics	16	1.3
**Supplements**	763	61.8
Vitamin C	715	57.9
Zinc supplements	366	29.6
Vitamin D	267	21.6
Vitamin E	128	10.4
Other supplements	24	1.9
Steroids	4	0.3

* Multiple responses allowed

**Bivariate analysis**: analysis showed that older people (p=0.002), people who had at least tertiary education (p=0.001) were more likely to wash their hands, older people were also more likely to maintain physical distancing (p=0.011). The study also showed that use of hand sanitizers was associated with higher educational status (p=0.001). There was no significant association between wearing of face masks and other variables. Higher drug use and herbal therapies were found to be significantly higher in people who were exposed to a suspected case of COVID-19 (P <0.001 and 0.011 respectively). There was no association between supplement use and previous exposure.

## Discussion

This study investigated behavioural adaptation and the use of drugs and other remedies for COVID -19 prevention among Nigerian adults. There was a high adherence to behavioural recommendations among the population and a high propensity for drug misuse. A significant proportion (above 90%) of the study population used face masks, washed their hands regularly and used sanitizers. This is in consonance with an internet survey of behavioural adherence from 8 countries (France, Germany, Poland, Russia, Spain, Sweden, U.K., U.S.) which reported adherence to behavioural measures at 91.7% [15]. Similar patterns of high adherence to behavioural changes were reported in a study in Singapore [16]. In contrast, adherence to behavioural measures was lower in Democratic republic of Congo (DRC), another African country in comparison to Nigeria. The study, which carried out two online surveys showed level of adherence to regular handwashing at 85% and 77%, respectively, wearing of facemasks at 41.4% and 69%, respectively and physical distancing at 58% and 43.4%, respectively [17]. Low compliance to behaviuoral recommendation were also reported in a study involving six other countries; China, Italy, Japan, Korea, the UK, and the USA [18].

From our findings, people are less likely to adhere to social distancing compared to other measures, this was replicated in the study by Ditekemena and colleagues (17). Another study which explored the factors responsible for non-adherence to social distancing showed that the respondents´ need to interact to avoid loneliness, as well as to perform important tasks were the major facilitator of poor adherence to social distancing [19]. These behavioural measures are infection control practices which are aimed at reducing the rate of transmission of pathogens by direct contact or through droplets spread, but variables like time to implement preventive measures and correct use of preventive measures are known to affect the outcome of these [2,20]. It has been shown that early introduction of facemasks is more effective in prevention compared to adoption of its use at the peak of transmission [2]. It is expected that countries that introduced these measures earlier will likely have lower rates of transmission. Correct use of preventive measures is also important, an observational study carried out in a public space over a period of three days reported that about 66.5-73.6% of the population used facemasks, although more than half of them wore the facemasks incorrectly [20].

Since the outbreak of the pandemic, several preventive therapies have been adopted by individuals to reduce their likelihood of being infected. In this study, common measures adopted include the use of drugs or herbal based therapy in 69%, consumption of fruits 42.5%, steam inhalation in 32.2% and drinking of hot water in 31.6% of the participants. Little is known about the efficacy of these measures, while measures like consumption of fruits may be beneficial in terms of improving immunity and general wellbeing, other measures such as use of drugs or herbal based therapy and steam inhalation calls for caution because of safety issues. A case report suggested that steam inhalation may be promising for the prevention of COVID-19 [21] but the high risk of dissemination of infection through droplet spread is a bigger concern with its use [22]. Of the methods adopted, the effect of drugs has been relatively well researched, several *in silico, invivo* and clinical trials have been carried out on different drug candidates but results have been largely inconclusive [23-26]. Despite the inconsistent or lack of evidence on the efficacy of drugs and herbal based therapies in prevention of COVID -19, these agents are being used to prevent or treat COVID both in hospital and home setting [12].

A large proportion (69%) of the study population had taken at least one drug or herbal based therapy for the prevention of COVID-19. The most used group of drugs was antibiotics which was consumed in about one fifth of the participants, ivermectin; an anti-helminthic agent and antimalarial were used in about a tenth of the participants respectively. A similar pattern of drug use was observed in a Bangladesh study where self-medication was observed in 88.3% and the most frequently used prescription-only drugs were antibiotics (which included azithromycin (54.2%), doxycycline (40.3%) and ivermectin (77.2%) [13]. The major determinant of drug or herbal uses was a history of previous exposure to a COVID-19 patient although about one out of three participants who had no previous contact with a COVID-19 positive patient took a drug, with some of the drugs being prescription only medications. The pandemic has brought to fore the inherent weak drug regulatory policies in developing countries where people can easily obtain medications without a prescription [13].

The pattern of antibiotic use is rather worrisome because the uncontrolled use and misuse of these classes of drugs have been shown to be major catalysts for the development of antimicrobial resistance. While antibiotic use for the treatment of concomitant bacterial coinfection in COVID-19 is not debatable, the use as prophylaxis for bacterial coinfection has not shown any clear benefit since there is no evidence suggesting higher rates of bacterial infections. A meta-analysis comparing the prevalence of bacterial/fungal co-infection in COVID-19 versus non COVID-19 admissions reported 8% and 11% respectively [27]. Another study which evaluated the prevalence of bacterial infection in COVID-19 patients showed only 3.5% had a bacterial co-infection before hospitalization and 14.3% secondary infection while hospitalized [28]. Antibiotics were used in monotherapy and combination therapy in this study, suggestive its use as an antiviral preventive therapy for COVID-19. Azithromycin was the most used antibiotic and the most investigated drug in COVID research. *In silico* and *in-vitro* activities have suggested antiviral activities but this has not been consistent in human studies [23,24].

High rates of prescription and use of antibiotics have also been reported in other studies. A meta-analysis also showed that 72% of patients reported receiving antimicrobial therapy despite a low rate of infection among the participants [27]. Another study reported that antimicrobial use was highly prevalent among suspected COVID-19 in-patients in Bangladesh with 77% mild and 94% moderately ill patients received antibiotics on admission [29]. Over the past few years, there has been a call to enforce antimicrobial stewardship in order to combat the looming pandemic of antimicrobial resistance [30]. Antibiotic resistance is a public health problem and a global issue, the uncontrolled use of antibiotics during the COVID -19 period may redefine the prevalence of antibiotic resistance globally. The use of ivermectin, an anti-helminthic agent has also been associated with drug resistance. Resistance to ivermectin has also been documented in veterinary medicine and may directly affect humans because of the possibility of spread of drug resistant parasites to humans, and indirectly by causing food shortage [31]. Current trends in ivermectin use may worsen the resistance. Although antimalarial drugs were in common use as prophylaxis in this study, current drugs recommended for therapy were used only in a small proportion of participants. This relatively low frequency of use of current antimalarial drugs portends a lower risk of drug resistance, nevertheless the possibility still remains. There is a need for urgent intervention for the new pattern of indiscriminate use of antibiotics in the COVID-19 era. The antimalarial drugs; hydroxychloroquine and chloroquine are not currently recommended as first line drugs for the management of malaria infection, so the major issue with their use borders on efficacy, adverse effect profile and drug shortage. The use of chloroquine and hydroxychloroquine as post exposure prophylaxis and treatment of COVID-19 has been a subject of intense debate globally. Hydroxychloroquine and chloroquine were initially approved under emergency use authorization for the treatment of COVID-19 based on an anecdotal report of its efficacy, but the decision was later rescinded because of non-convincing evidence of its efficacy and safety issues [26,32,33]. Several other studies have documented conflicting evidence of efficacy as prophylaxis or treatment either as monotherapy or in combination with other drugs [24-26,34]. A study investigating the use of hydroxychloroquine as a pre-exposure prophylaxis in healthcare workers did not show efficacy [35]. Hydroxychloroquine and chloroquine are used for the management of autoimmune diseases like rheumatoid arthritis, a major consequence of their increased uptake is drug shortage which has been reported in a large number of patients [33,36].

Drug supplements ware commonly used among participants, about two third of the population studied were on at least one supplement. A balanced nutrient is essential for normal physiological function and deficiency states are associated with disease. A study reported that reduced levels of vitamins A, E, B6, B12, D, zinc and selenium were associated with higher risk of infection and severe outcomes in COVID-19 patients [37]. Antiviral effects have also been reported for vitamin C and selenium [37,38]. This however has not been replicated in large clinical trials; therefore it has been suggested that vitamins, minerals, fatty acids and herbal based substances like black seeds, garlic, ginger should be used in combination with other drugs with stronger anti-viral effects to combat infection [39].

Like other drugs, the use of supplements has potentials for causing adverse effects when excessively high doses are used or because of drug-drug, drug-food and drug-disease interactions. An average enrollee in this study used more than 2 drugs thus increasing potentials for adverse effects. Drug toxicity has been reported with higher doses of vitamins and minerals because they are required in small doses [40]. There was a relatively low uptake of herbal based remedies in the population. Only about 10% of the population used agents like garlic, ginger, black seeds, and other plant products. The relatively low use may be associated with higher educational status of the population studied (Table 1). There is paucity of data on the effectiveness of herbal remedies and their adverse effect profile in COVID-19. This was an internet-based survey and we recognised that the result obtained from this study was obtained from the highly educated fraction of the society since more than 90% percent had tertiary education and may not reflect the behaviour of the entire populace. A follow up study in less educated populace may help to identify the effect of education on the variables studied.

## Conclusion

This is one of the few studies that evaluated adoption of infection control practices and drug uptake patterns in COVID-19 era in our environment. The response to recommended preventive measure was good among the population, but the population had a high propensity for drug misuse. The widespread misinformation from the media and the conflicting reports from poorly designed clinical trials might have contributed to the widespread drug misuse in the population [13]. There is a need to educate the public on drug misuse and its implications.

### What is known about this topic

- Behavioural interventions like social distancing, use of facemasks and hand washing are protective against the spread of infection but the level of adherence to behavioural changes is unknown in our population;
- Several drugs have been proposed as possible treatment for COVID-19, but the pattern of drug uptake among the population is unknown.

### What this study adds

- This study showed a high level of adherence to behavioural protocols in Nigeria at the period of research;
- Drug misuse was common, antibiotics misuse rate was high among the population.
